# Preparation, Characterization, and Release Kinetics of Chitosan-Coated Nanoliposomes Encapsulating Curcumin in Simulated Environments

**DOI:** 10.3390/molecules24102023

**Published:** 2019-05-27

**Authors:** Mahmoud Hasan, Kamil Elkhoury, Cyril J. F. Kahn, Elmira Arab-Tehrany, Michel Linder

**Affiliations:** Laboratoire D’ingénierie des Biomolécules, Université de Lorraine, EA 4367, France; mahmoud.hasan@univ-lorraine.fr (M.H.); kamil.elkhoury@univ-lorraine.fr (K.E.); cyril.kahn@univ-lorraine.fr (C.J.F.K.)

**Keywords:** chitosan, nanoliposomes, curcumin, release kinetics, gastrointestinal environment

## Abstract

Curcumin, a natural polyphenol, has many biological properties, such as anti-inflammatory, antioxidant, and anti-carcinogenic properties, yet, its sensitivity to light, oxygen, and heat, and its low solubility in water renders its preservation and bioavailability challenging. To increase its bioaccessibility, we fabricated nanoliposomes and chitosan-coated nanoliposomes encapsulating curcumin, and we evaluated the systems in terms of their physicochemical characteristics and release profiles in simulated gastrointestinal mediums. Chitosan-coating enhanced the stability of nanoliposomes and slowed the release of curcumin in the simulated gastrointestinal (GI) environment. This study demonstrates that nanoliposomes and chitosan-coated nanoliposomes are promising carriers for poorly soluble lipophilic compounds with low oral bioavailability, such as curcumin.

## 1. Introduction

Curcumin, extracted and purified from ground rhizomes of *Curcuma longa*, presents antioxidant [1,2,3], wound healing [4,5], anticancer [6,7,8], antimicrobial [9,10,11], and anti-inflammatory [12,13,14] properties. However, studies showed that this natural remedy presents low bioavailability as extremely low serum concentrations have been detected in the human serum after oral administration [15,16,17]. This can be attributed to the poor aqueous solubility of curcumin, fragmentation at physiological pH, and rapid hydrolysis [18].

Various delivery systems have been proposed to improve the bioavailability and the therapeutic efficacy of curcumin, such as polymer nanoparticles [19,20], lipid-based nanoparticles [21,22], and inorganic nanoparticles [23]. Liposomes are one of the oldest and most widely used lipid-based delivery carriers, due to their low toxicity, their biocompatibility, and non-immunogenicity [24]. These nanovehicles are broadly used to overcome physiological barriers and deliver drugs to cells and tissues [25,26]. They are able to encapsulate poorly soluble molecules and enable their administration in aqueous mediums [27]. These features are presented by liposomes thanks to their main constituents, phospholipids, which are responsible for the liposome structure resemblance to cell membranes [28,29]. Phospholipids are amphiphilic molecules composed of a hydrophilic head and a hydrophobic tail. Their amphiphilic property allows the self-sealing of liposomes, which renders them an ideal delivery system in aqueous media and has various applications in the cosmetic [30], food [31], pharmaceutical [32], and tissue engineering [33,34] fields.

Lecithin, from which liposomes can be produced, possesses two long hydrocarbon chains and is a major component of lipid bilayers in cell membranes. Moreover, due to its biodegradability, lecithin is considered as an ideal biological surfactant [35]. Numerous in vivo studies have proved that the n-3 series polyunsaturated fatty acids (PUFAs), especially docosahexaenoic acid (DHA, 22:6 n-3) and eicosapentaenoic acid (EPA, 20:5 n-3), are important to various physiological processes, which are present in high concentrations in lecithin from the salmon head [36,37,38,39].

Destruction in the gastrointestinal tract by pH, pancreatic lipase, and bile salts, presents the biggest challenge for liposomal delivery systems [40]. Previous studies have analyzed the effects of coating liposomes with a polymeric membrane to minimize these effects [41,42]. Several studies have investigated the coating of liposomes with chitosan [42,43,44]. The positively charged chitosan forms an ionic bond with negatively charged liposomes to create the chitosan-coated liposomes [45]. Coating these nanovesicles with chitosan renders them mucoadhesive and increases their stability [45,46]. By combining chitosan and liposomal characteristics, specific, prolonged, and controlled release may be achieved [47]. Because of its bioadhesivity, low toxicity, permeation enhancing properties, hydrophilicity, biocompatibility, and biodegradability, chitosan has received substantial attention in novel drug delivery systems, aimed at improving the bioavailability of drugs at the targeted site of absorption [48].

The present study focused on the preparation and physicochemical characterization of chitosan-coated nanoliposomes produced from salmon-extracted lecithin. Additionally, and more importantly, the release kinetics of encapsulated curcumin inside the coated system in simulated gastric and intestinal digestion environments was investigated.

## 2. Results and Discussion

### 2.1. Fatty Acid Analyses

Table 1 presents the purified salmon phospholipids’ main fatty acid compositions. The total polyunsaturated fatty acids in salmon phospholipids were predominate (48.72%). This percentage was followed by the saturated fatty acids percentage (27.67%). C20:5 n-3 (8.83%) and C22:6 n-3 (28.15%) in the polyunsaturated fatty acids class, C16:0 (29.33%) in the saturated fatty acids class, and C18:1 n-9 (19.12%) in the monounsaturated fatty acids class were the main proportions of fatty acids. The ratio of n-3/n-6 was 8.73, and DHA/EPA was 3.19.

### 2.2. Lipid Classes

Thin-layer chromatography (Iatroscan) was used to separate the lipid classes of salmon phospholipids. The major class of phospholipids was phosphatidylcholine (40.49%), followed by phosphatidylinositol (15.25%), and phosphatidylserine (7.95%). It is interesting to note that phospholipids can enhance drug flux; they are called a sorption promoter [49]. The percentage of polar lipid in salmon phospholipids was 100%, whereas it did not contain any triglycerides or other nonpolar lipids.

### 2.3. Physicochemical Characterization

Lipid composition and preparation methods can affect nanoliposome particles size [50]. Immediately after their preparation, nanoliposomes average particle size was measured. The mean size of the uncoated nanoliposomes was 115.8 ± 3.5 nm and increased to 330.4 ± 5.1 nm after coating with chitosan. The size of the nanoliposomes encapsulating curcumin (114.9 ± 1.8 nm) was equal to the size of empty nanoliposomes. Similarly, chitosan-coated curcumin-loaded nanoliposomes size (324.7 ± 2.2 nm) was similar to the unloaded coated nanoparticles size. This suggested that the curcumin encapsulation had minimal effect on the coated and uncoated nanoparticle size.

**Figure 1:**
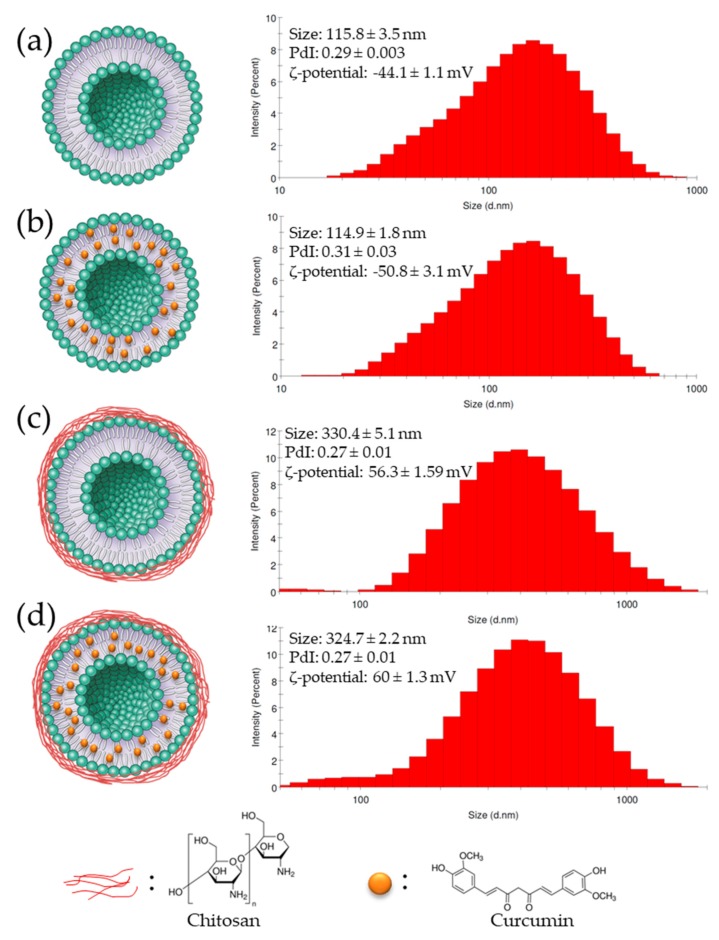
Schematic and physicochemical characterization of (**a**) nanoliposomes, (**b**) curcumin-loaded nanoliposomes, (**c**) chitosan-coated nanoliposomes, and (**d**) curcumin-loaded nanoliposomes coated with chitosan.

The polydispersity index (PDI) is a dimensionless measure of particle size distribution, also measured by dynamic light scattering (DLS) [51]. Figure 1 shows that PDIs of all formulations were between 0.27 and 0.31, which indicates that particles had a controlled size distribution and a narrow dispersity.

ζ-potential measurements characterize the particles’ surface electrical charge [52]. The greater the ζ-potential magnitude, the greater the repulsion between particles, and thus the, more stable the colloidal dispersions [53]. Coating the empty and loaded nanoliposomes with chitosan (Figure 1) shifted the ζ-potential from negative (−44.1 ± 1.1 mV and −50.8 ± 3.1 mV) to positive values (+56.3 ± 1.59 mV and +60 ± 1.3 mV), respectively. Chitosan-coating led to a higher positive ζ-potential magnitude, which suggests that the coating increased the stability of nanoliposomes

The polymer-liposome interactions caused an increase in particle size and ζ-potential of nanoparticles, which reflects several changes in their surface properties.

With respect to the formulation stability with time, no significant variation in particle size of uncoated and chitosan-coated nanoliposomes, encapsulating curcumin or not, was observed over 30 days of incubation at 4 and 37 °C.

### 2.4. Entrapment Efficiency

Curcumin entrapment efficiency in salmon nanoliposomes was 78.2 ± 0.5%, which was greater than our previous results (67.3 ± 1.1% [8]), due to a change in the loading method. In this study, we loaded the nanoliposomes using the thin film method, where we solubilized the phospholipids and curcumin in a chloroform/methanol mixture (2:1 *v*/*v*), before evaporating it using a Rotavapor. This increased the distribution of curcumin in the phospholipids and hence increased the entrapment efficiency. Moreover, the encapsulation efficiency of curcumin significantly increases when nanoliposomes are coated with chitosan, with an entrapment efficiency of 95.2 ± 1.2%.

### 2.5. In Vitro Drug Release

#### 2.5.1. Release in PBS Solution

**Figure 2:**
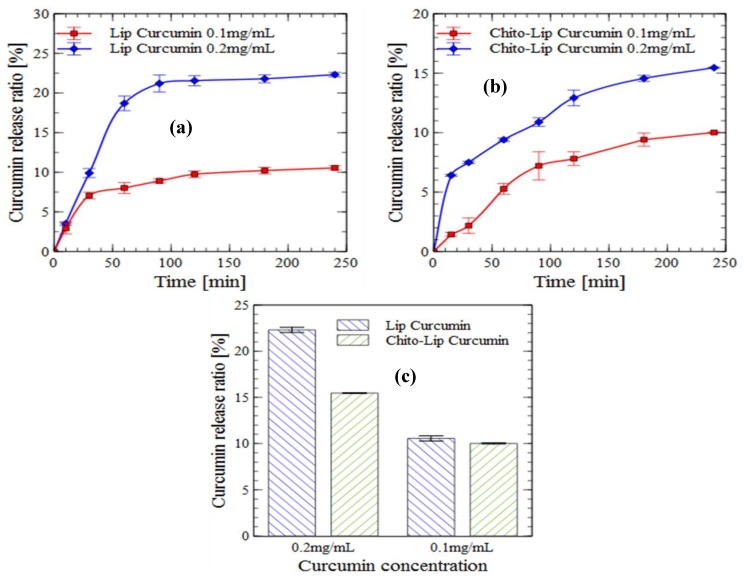
In vitro release (PBS solution) of curcumin encapsulated in nanoliposomes (**a**) and chitosan-coated nanoliposomes (**b**) (values reported are mean ± SD; *n* = 3), and the proportions of released curcumin from nanoliposomes and chitosan-coated nanoliposomes after four hours of incubation (**c**).

Curcumin encapsulated in uncoated and chitosan-coated nanoliposomes was incubated at 37 °C in PBS, and it’s in vitro release behavior was studied over four hours and is presented as a cumulative release percentage in Figure 2. A biphasic release profile was observed for all formulations. A biphasic release profile is composed of an immediate drug release followed by a sustained release [54]. The biphasic delivery system helps in overcoming multiple dosing regimen problems [55].

In the case of uncoated nanoliposomes charged with 0.2 mg/mL curcumin, a fast release (18%) was observed at the end of the first hour, followed by a sustained release (22.3%) over the remaining three hours. The initial burst release decreased, releasing only 10% of the encapsulated drug for a curcumin concentration of 0.2 mg/mL, and the sustained release of curcumin from the colloid particles was more pronounced.

The initial burst release is caused by the release of the drug entrapped near the surface, whereas the sustained release is the result of the diffusion and release of the drug from the nanoliposome core. In the case of coated nanoliposomes, the biphasic release behavior is affected by the presence of the chitosan layer, formed via electrostatic interactions between the positive ammonium groups of chitosan and the negatively charged phosphate groups of the nanoliposomes, as previously determined by Fourier-transform infrared spectroscopy (FTIR) analysis [44]. This coating layer will probably form a diffusion obstacle for the drug released from the surface, resulting in a slower release [56]. Furthermore, the coating of nanoliposomes with chitosan causes a decrease in membrane fluidity, which is crucial in the release behavior of the entrapped drug [44,57].

In respect to the concentration of loaded curcumin, we observed that the release profile of curcumin was almost similar for both concentrations of 0.1 and 0.2 mg/mL of the chitosan-coated particles. However, their difference in release profiles was noticeable for uncoated nanoliposomes. Therefore, these results imply that the release profile of core material from the nanocarrier systems is significantly affected and can be tuned by applying a polymer coating.

#### 2.5.2. Gastric Digestion

**Figure 3:**
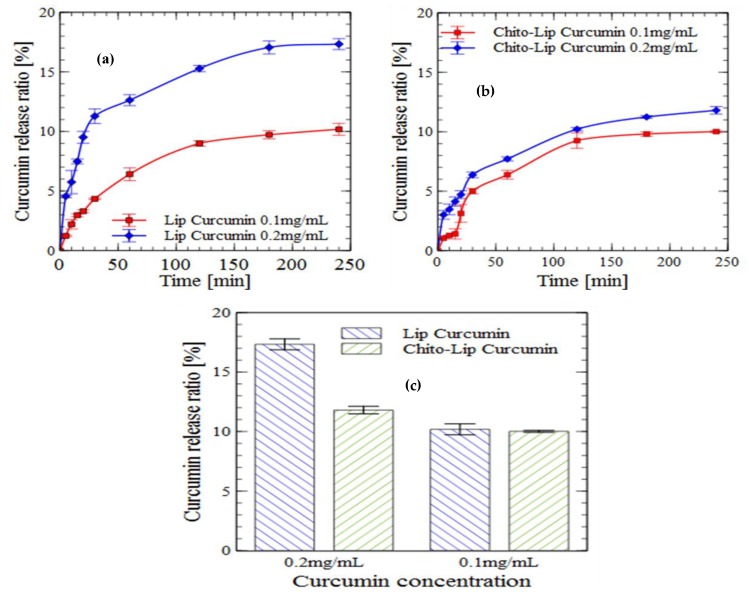
In vitro release (gastric digestion) of curcumin encapsulated in nanoliposomes (**a**) and chitosan-coated nanoliposomes (**b**) (values reported are mean ± SD; *n* = 3), and the proportions of released curcumin from nanoliposomes and chitosan-coated nanoliposomes after four hours of incubation (**c**).

The in vitro release profiles of curcumin from uncoated nanoliposomes and chitosan-coated liposomes in a simulated gastric digestion environment are presented in Figure 3. The results indicated that encapsulated curcumin was well protected by nanoliposomes from pepsin action. Over 80% of curcumin was retained in uncoated nanoliposomes and about 90% for the chitosan-coated liposome during simulated gastric digestion (four hours). Drug release percentage in the simulated gastric fluid (SGF) decreased for chitosan-coated nanoliposomes, which is a desirable attribute to protect the bioactive molecule from the severe gastric environment [58,59].

Release profiles of curcumin during gastric incubation were almost similar to their profiles in the PBS solution, with a slight decrease especially for uncoated nanoliposomes, for which the maximum curcumin released after four hours of incubation was 23.3% in PBS and 17.33% in the simulated gastric environment.

#### 2.5.3. Intestinal Digestion

The curcumin release rate from loaded-nanoliposomes was higher in simulated intestinal fluid (SIF), a mixture of pancreatin and bile salts, than in SGF, which was also found in previous studies [60,61,62]. Liu et al. suggested that, under SIF conditions, this behavior is caused by the pancreatic enzyme that disrupts the liposomal membrane [62].

**Figure 4:**
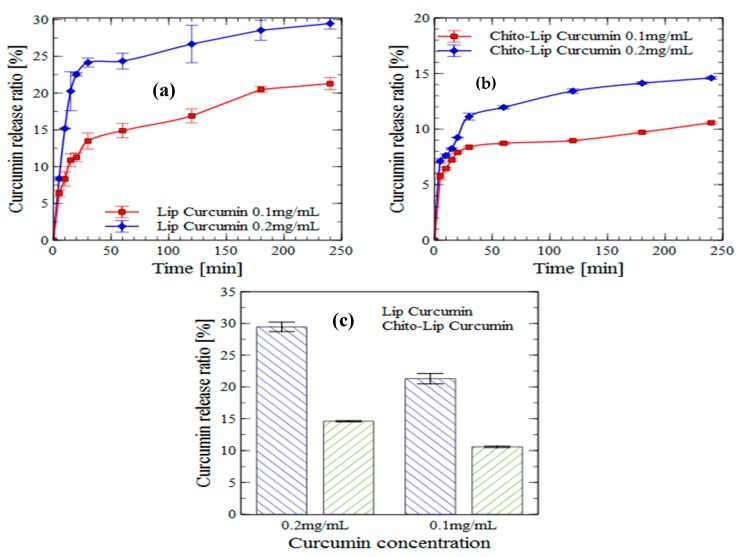
In vitro release (intestinal digestion) of curcumin encapsulated in nanoliposomes (**a**) and chitosan-coated nanoliposomes (**b**) (values reported are mean ± SD; *n* = 3), and the proportions of released curcumin from nanoliposomes and chitosan-coated nanoliposomes after four hours of incubation (**c**).

A biphasic release was also observed, especially in the case of uncoated nanoliposomes charged with 0.2 mg/mL curcumin, presenting a rapid release of about 24.5% at the end of the first 30 min, that was followed by a sustained release of around 29.5% over four hours (Figure 4).

Curcumin was released in a controlled manner for longer durations. These results suggest that loaded nanoliposomes can be parenterally administered, thus avoiding the harsh gastrointestinal route and control the release of the low bioavailable curcumin.

To improve their stability and to target specific areas in the body, the liposomal surface can be modified [63,64]. One possible modification is the coating with chitosan, which can increase nanoliposomes stability in SGF and SIF, and improve their encapsulated drug solubility and mucoadhesive properties [65].

Determining curcumin release from chitosan-coated liposome in SIF is challenging because of the precipitation of chitosan at pH 6.8 [66]. For this reason, we added 0.5% Tween-80 in SIF in order to solubilize the released curcumin during the digestion.

The curcumin release rate from all nanoliposome formulations was higher in the simulated intestinal fluid than in the simulated gastric fluid. Moreover, the release was much lower when the chitosan coating was applied, which might be due to the fact that polysaccharides have the capacity to inhibit lipid digestion [67,68].

Despite the chitinase and chitotriosidase activities of the pancreatic enzyme, it is better to complete the simulated digestion in the presence of other enzymes, such as beta-glucosidase and rat or human colonic enzymes, as chitosan will be degraded predominantly by lysozymes and bacterial enzymes in the colon [69,70,71,72].

## 3. Materials and Methods

Chitosan, with a deacetylation degree up to 75% prepared from shrimp shells, curcumin, boron trifluoride (14% in methanol), acetonitrile (≥99.9%), chloroform (≥99.9%), methanol (≥99.9%), and hexane (≥99.9%) were all purchased from Fisher (France) and Sigma-Aldrich (France). Acetic acid (≥99.8%) was supplied by Prolabo-VWR. *Salmo salar* were enzymatically hydrolyzed to obtain lecithin in an organic solvent-free extraction, as previously described by Linder et al. [73].

### 3.1. Fatty Acids Composition

Fatty acid methyl esters (FAMEs) were analyzed as previously described [74]. FAMEs separation was carried out on gas chromatography (Perichrom, Saulx-lès-Chartreux, France). Injector and detector temperatures were fixed at 250 °C. The column temperature was initially set at 120 °C for three min, then it was increased to 180 °C at a rate of 2 °C·min^−1^ and kept at 220 °C for 25 min. Standard mixtures (Supelco, Sigma–Aldrich, Bellefonte, PA, USA) were used to categorize fatty acids. All runs were performed in triplicate.

### 3.2. Lipid Classes

Lipid classes of salmon phospholipids were determined by an Iatroscan (MK-5 TLC-FID, Iatron Laboratories Inc., Tokyo, Japan) as described in detail previously [8]. Two migrations were executed to characterize the proportion of polar and neutral lipid fractions. Area percentages were shown as the average of three repetitions.

### 3.3. Nanoliposomes Preparation and Coating

Loaded nanoliposomes and chitosan-coated nanoliposomes have been prepared as described previously [44]. In a completely dry round-bottomed flask, 1.5 g of salmon phospholipid dissolved in 30 mL of chloroform and 10 mg of curcumin dissolved in 15 mL of methanol were mixed. Organic solvents were completely evaporated using a Rotavapor under vacuum, and a thin lipid film was formed on the flask wall. 47.5 mL of ultrapure water was then added, and the suspension was agitated for four hours under nitrogen. To produce nanoliposomes, the suspension was sonicated at 40 kHz for five min (1 s on, 1 s stop) in an ice bath.

To form the coated nanoliposomes, 0.5 g of chitosan and 0.5 mL of acetic acid were added to the solution already prepared before sonication. The suspension was then agitated under the same conditions for 4–5 min. Sonication at 40 kHz for five min (1 s on, 1 s stop) in an ice bath was first applied, followed by homogenization using a high-pressure homogenizer (EmulsiFlex-C3). The mixture was introduced under a pressure of 1500 bar in quantities of 50 mL for 7–8 cycles.

Uncoated and Chitosan-coated nanoliposome samples were stored in glass bottles in the dark at 4 and 37 °C.

### 3.4. Size and ζ-Potential Measurements

The size, PDI, and ζ-potential of nanoliposomes were measured by DLS (Malvern Zetasizer Nano ZS, UK) following the dilution of samples (1:200) using ultrapure water. Samples were analyzed in standard capillary electrophoresis cells to measure their ζ-potential. Size and ζ-potential measurements were studied with an absorbance of 0.01, a fixed scattering angle of 173°, a refractive index of 1.471, and at 25 °C. Measurements were completed in triplicate.

### 3.5. Stability of Nanoliposomes and Chitosan-Coated Nanoliposomes

Uncoated and chitosan-coated nanoliposomes, both empty or encapsulating curcumin, were stored in a drying room for 30 days at 4 and 37 °C. Physicochemical characterization of all formulations was examined every three days.

### 3.6. Entrapment Efficiency

The percentage of curcumin encapsulated was determined by centrifuging the coated and uncoated nanoliposomes encapsulating curcumin at 9000× *g* for 15 min. After centrifugation, the supernatant was dissolved in methanol to erupt the nanoliposomes and release the entrapped curcumin. The concentration of encapsulated curcumin was measured by an HPLC system (Shimadzu, Kyoto, Japan) at 425 nm equipped with a Zorbex SB-C18 column (5 µm, 4.6 mm × 250 mm). The suspension was quantified in an isocratic mode using acetonitrile (*v*/*v*, 65%), acetic acid 2% (*v*/*v*, 30%), and methanol (*v*/*v*, 5%) at a flow rate of 0.5 mL·min^−1^. Experiments were performed in triplicate.

Entrapment efficiency was calculated according to the following equation:

EE (%) = (Initial drug − Free drug)/(Initial drug) × 100,(1)

### 3.7. In Vitro Drug Release

#### 3.7.1. Release in PBS Solution

Curcumin released percentages were determined as previously described [75]. In brief, nanoliposomes encapsulating curcuminoids were dispersed in PBS (pH 7.4) at a ratio of 1:2 and free curcuminoid molecules were removed via centrifugation at 9000× *g* for 15 min. All formulations were equally separated into 24 aliquots of 1.8 mL in Eppendorf tubes and stored in a water bath at 37 °C. Because of the low curcumin solubility in an aqueous medium, released crystals precipitated at the bottom of the tubes. The sample was centrifuged at 9000× *g* for 15 min to separate the released curcuminoid crystals from the loaded nanoliposome, at predetermined time intervals. After centrifugation, released curcumin crystals aggregated in the pellet and the supernatant containing the loaded nanoliposomes was assayed by HPLC at 425 nm after dissolution with methanol to determine the amount of encapsulated curcuminoids. All experiments were carried out in triplicate.

The percentage of released curcuminoids was determined using the formula:

% Drug release = (Released Curcuminoids/Encapsulated Curcuminoids) × 100,(2)

#### 3.7.2. Simulated Gastric Digestion

SGF which virtually mimics the stomach conditions was constituted as described earlier with slight modification [76]. Briefly, after removing free curcuminoids molecules via centrifugation at 9000× *g* for 15 min. Samples were added to the SGF (0.2 wt.% NaCl, pepsin 0.32 wt.% (from porcine stomach mucosa), pH 2), and the pH was adjusted to 2 with 0.5 M HCl.

Curcumin-loaded nanoliposomes (adjusted to pH 2) were mixed with SGF at a ratio of 1:2 in a flask. The mixture was placed in a shaking water bath (170 rpm) at 37 °C. Samples were collected at different time intervals, and the digestion was stopped by placing the Eppendorf tubes in cold water for 15 min, then the released curcumin was measured using the same method for PBS explained earlier. All experiments were carried out in triplicate.

#### 3.7.3. Simulated Intestinal Digestion

The same preparation method of SIF described before was used with slight modifications [77,78]. Part of the juice obtained after digestion in SGF for two hours, which is the normal duration of GI transit, was incubated in SIF containing (30 mM CaCl2, 1.0 wt.% Pancreatin (from porcine pancreas), 0.5 wt.% Bile salts, and 25 mM potassium dihydrogen phosphate, pH 6.8). The pH was adjusted to 6.8 with 0.5 M NaOH.

The mixture was placed in a shaking water bath (170 rpm) at 37 °C. The samples were pipetted out from the flask at predetermined time intervals, and the Eppendorf tubes were immediately put in an ice-cold water bath for 15 min to stop the further hydrolysis. Then, the released drug was determined using the same method for PBS explained above, and all the experiments were carried out in triplicate.

In the case of chitosan-coated liposomes, the solution was added to SIF 0.5% (*w*/*v*) Tween-80 to solubilize free-released curcumin [63], then the same digestive conditions were followed. The samples were centrifuged first at 9000× *g* for 15 min to eliminate the precipitated particles, then the supernatant which contains the free curcumin dissolved in Tween-80 solution was separated by centrifugation at 3300× *g* for 20 min using a membrane filter (Centrisart^®^ I, MWCO 100 kDa; Sartorius, GmbH Germany) [79].

## 4. Conclusions

Curcumin is highly unstable and hydrophobic, which makes its oral administration challenging. For this reason, curcumin was encapsulated in nanoliposomes and chitosan-coated nanoliposomes. The ζ-potential of the empty and loaded nanoliposomes changed to an increasingly positive value for the chitosan-coated compared to a negative value for the uncoated nanoliposomes.

The results presented in this study proved that curcumin was released in a controlled manner from all nanoliposome formulations under simulated digestion. Moreover, the curcumin release rate from all nanoliposome formulations was higher in SIF than in SGF.

Chitosan-coated nanoliposomes exhibited a more stable and prolonged release of curcumin in comparison to uncoated nanoliposomes, especially in SGF. The results, presented in this study, demonstrate that chitosan-coated nanoliposomes might be an efficient drug delivery system for the oral delivery of curcumin.

## Figures and Tables

**Table 1:** Fatty acid compositions, after purification by acetone precipitation, of salmon phospholipids.

Fatty Acids	Salmon Phospholipids % SD	
C14	2.24	0.04
C15	0.28	0.00
C16	19.33	0.21
C17	0.55	0.02
C18	4.47	0.03
C20	0.22	0.02
C22	0.58	0.01
SFA	27.67	
C16:1n7	1.83	0.02
C18:1n9	19.11	0.29
C20:1n9	0.28	0.01
C22:1n9	2.39	0.00
MUFA	23.61	
C18:2n6	4.41	0.07
C18:3n3	1.98	0.05
C20:4n6	2.84	0.04
C20:5n3(EPA)	8.83	0.02
C22:5n3	2.51	0.10
C22:6n3(DHA)	28.15	0.19
PUFA	48.72	
n-3/n-6	10.06	
DHA/EPA	3.19	

## Abbreviations

SFA	Saturated fatty acids;
MUFA	Monounsaturated fatty acids;
PUFA	Polyunsaturated fatty Acids;
EPA	Eicosapentaenoic acid;
DHA	Docosahexaenoic Acid;
n-3	*Omega-3 fatty acids;*
n-6	Omega-6 fatty acids.
